# Grassland degradation affected vegetation carbon density but not soil carbon density

**DOI:** 10.1186/s12870-024-05409-6

**Published:** 2024-07-29

**Authors:** Chan Zhou, Hainan Xia, Tingting Yang, Zhuo Zhang, Guobin Zheng

**Affiliations:** 1 School of Life Science, Liaoning University, Shenyang, 110036 People’s Republic of China; 2 School of Life Science and Bioengineering, Shenyang University, Shenyang, 110044 People’s Republic of China

**Keywords:** Grassland degradation, Carbon density, Carbon storage, Meadow steppe, Northwest Liaoning

## Abstract

**Background:**

With the profound changes in the global climate, the issue of grassland degradation is becoming increasingly prominent. Grassland degradation poses a severe threat to the carbon cycle and carbon storage within grassland ecosystems. Additionally, it will adversely affect the sustainability of food production. The grassland ecosystem in the northwest region of Liaoning Province, China, is particularly vulnerable due to factors such as erosion from the northern Horqin Sandy Land, persistent arid climate, and issues related to overgrazing and mismanagement of grassland. The degradation issue is especially pronounced in this ecological environment. However, previous research on the carbon density of degraded grasslands in Northeast China has predominantly focused on Inner Mongolia, neglecting the impact on the grasslands in the northwest of Liaoning Province. Therefore, this experiment aims to assess the influence of grassland degradation intensity on the vegetation and soil carbon density in the northwest of Liaoning Province. The objective is to investigate the changes in grassland vegetation and soil carbon density resulting from different degrees of grassland degradation.

**Methodology:**

This study focuses on the carbon density of grasslands at different degrees of degradation in the northwest of Liaoning Province, exploring the variations in vegetation and soil carbon density under different levels of degradation. This experiment employed field sampling techniques to establish 100 × 100 m plots in grasslands exhibiting varying degrees of degradation. Six replications of 100 × 100 m plots per degradation intensity were sampled. Vegetation and soil samples were collected for analysis of carbon density.

**Results:**

The results indicate that in the context of grassland degradation, there is a significant reduction in vegetation carbon density. Furthermore, it was found that root carbon density is the primary contributor to vegetation carbon density. In comparison to mildly degraded grasslands, moderately and severely degraded grasslands experience a reduction in vegetation carbon density by 25.6% and 52.6%, respectively. However, with regard to the impact of grassland degradation on soil carbon density, it was observed that while grassland degradation leads to a slight decrease in soil carbon density, there is no significant change in soil carbon density in the short term under the influence of grassland degradation.

**Conclusions:**

Therefore, grassland degradation has exerted a negative impact on aboveground vegetation carbon density, reducing the carbon storage of above-ground vegetation in grasslands. However, there was no significant effect on grassland soil carbon density.

## Background

Grassland ecosystems play a critical role in regulating the global carbon balance by storing a considerable amount of terrestrial carbon (C) [1], representing more than 34% of the total storage [2, 3]. Grasslands can be a significant soil carbon sink and store around one-third of the world's terrestrial carbon reserves [4, 5].Soil organic carbon stocks are even more extensive, contributing 20–30% of the total terrestrial carbon, while biomass carbon contributes 10% of the total terrestrial biomass carbon [6, 7]. Recent studies indicate that enhancing carbon input from grassland vegetation can increase underground biomass carbon input and enhance microbial activity. This, in turn, intensifies the decomposition of plant litter by microorganisms, leading to an enhanced storage of soil organic carbon [8–10]. Grassland vegetation and soil carbon pools are integral to ecosystem carbon cycling, serving as carbon sinks for grassland ecosystems [11–13]. However, in recent years, grassland degradation in China has varied significantly, with degradation affecting approximately 40% of the area [14–17]. Degradation is more prevalent in arid and semi-arid regions due to prolonged drought, significant changes in global climate, and increased human grazing activities [18, 19]. Grassland degradation results in a decline in vegetation productivity, affecting both plant carbon input and soil microbial decomposition activity. The reduction in soil surface cover further leads to the release of soil carbon into the atmosphere. Ultimately, this impacts the carbon storage in grassland vegetation and soil, causing a decrease in the carbon sequestration capacity of grasslands [20, 21]. Therefore, investigating the impact of grassland degradation at different levels on grassland vegetation and soil carbon density is a critical factor in assessing carbon cycling and atmospheric carbon fixation. Additionally, it can provide data support for the scientific management of grasslands.

Vegetation carbon is primarily introduced into ecosystems via vegetation photosynthesis. The density of vegetation carbon, which includes both above and below-ground carbon, is a critical factor influencing carbon sequestration in grassland ecosystems [21]. Additionally, it plays a role in controlling the residence time of carbon in both vegetation and soil pools. In recent years, the degradation of grasslands has resulted in a significant loss of carbon from grassland vegetation [22]. This degradation has caused a reduction in vegetation coverage and biodiversity, ultimately leading to a decrease in vegetation carbon stocks. Furthermore, the decrease in plant litter and the biomass of living plants (both above and below ground), specifically in terms of carbon content, constitutes two pivotal variables affecting the decline in soil carbon storage in degraded sandy grassland vegetation [23, 24]. In a study conducted on Xilinhot grassland, Inner Mongolia, indicated that total carbon stock in the degraded grassland decreased by 14% as degradation increased. The loss of vegetation biomass and significant reduction in carbon keep on capacity throughout the process of grassland degradation eventually led to the loss of vegetation carbon stocks and resulted in an unbalanced carbon budget [25]. Furthermore, the study on the effect of grazing-induced grassland degradation on vegetation biomass and soil organic carbon revealed that grasslands with high grazing intensity showed a decrease in vegetation biomass and soil organic carbon content. As grazing intensity increased, the effect on vegetation carbon became more pronounced [26]. However, there is limited research indicating the specific changes in grassland vegetation carbon density as degradation intensifies, as well as proposing corresponding strategies on how to address and improve this situation [27, 28]. So, studying the impact of various degrees of degradation on grassland vegetation carbon density is crucial for both the sequestration and input of vegetation carbon [29].

Soil serves as a vital carbon reservoir, storing approximately twice the amount of carbon as vegetation and the atmosphere combined. However, the majority of organic carbon in the soil is highly sensitive to land degradation [16, 30]. The potential for SOC sequestration in global grasslands ranges from 2.3 to 7.3 billion tonnes [31]of carbon dioxide equivalents annually (CO^2^e year^1^) for biodiversity restoration [32], 148 to 699 megatons of CO^2^e year^1^ for better grazing management [33], and 147 megatons of CO^2^e year^1^ for sown legumes in pasturelands [34]. As degradation intensifies, the organic carbon content in the soil significantly decreases, while soil compaction, hardness, and reduced water-holding capacity increase. In mixed agriculture and animal husbandry zones, degraded grasslands exhibit significantly lower soil quality than undegraded meadow grasslands and grasslands, with this phenomenon positively correlated with the carbon stock and grassland biomass of degraded grasslands [35]. For example. a study of degraded sandy grasslands in Horqin found that surface carbon accumulation in degraded grasslands had decreased significantly. Severely degraded and very severely degraded grasslands had reduced carbon stocks of 215% and 474%, respectively, compared to non-degraded grasslands, while moderately degraded grasslands had reduced carbon stocks of 111%. This reduction was primarily due to the loss of soil nutrients resulting from degradation [23]. Additionally, a research pointed out that degradation of grasslands not only caused the loss of organic carbon from soil, but also inorganic carbon, primarily in the deeper layers (30–70 cm) and topsoil (0–40 cm) [16]. The loss of soil carbon through degradation leads to increased CO_2_ emissions from soils, with subsequent impacts on global climate change [21]. Therefore, examining the effects of degradation on soil carbon density in grasslands is critical to enhancing grassland ecosystem productivity and understanding the carbon sequestration and storage capacity of grasslands.

Northwest Liaoning is a semi-arid area situated at the junction of the Horqin Sands and the Liaohe Plain. Due to the southward encroachment of the Horqin Sands and persistent droughts, the ecological environment in this region is highly delicate, and overgrazing has led to significant grassland degradation [36, 37]. As a result, there has been a decline in original plant species diversity and biomass of grassland vegetation, resulting in a sharp reduction in the area of natural grasslands [38]. These changes in plant composition and soil properties have led to a decrease in carbon stocks within grassland ecosystems, posing a severe threat to vegetation and soil carbon stocks due to increased CO_2_ release from vegetation and soils to the atmosphere [39, 40]. However, prior research on the ecological issues and changes in carbon density resulting from grassland degradation in northeastern China has mainly focused on the grasslands in Inner Mongolia [41–43]. Studies on the degradation of grasslands in the northwest of Liaoning Province have predominantly emphasized the impact of grassland degradation on vegetation diversity and the influence of dominant species on the carbon and nitrogen dynamics in degraded grasslands [44, 45]. There is limited research specifically addressing the concrete effects of grassland degradation on grassland vegetation and soil carbon density in this region. Thus, an investigation into vegetation and soil carbon density in degraded grasslands in northwest Liaoning is essential to quantify the carbon stocks of degraded grasslands in this region, cope with further degradation of grasslands, and maintain carbon balance.

Therefore, we conducted a study on vegetation and soil carbon density in different degraded grasslands in northwest Liaoning, aiming to elucidate the impact of the intensity of grassland degradation on grassland vegetation and soil carbon density. We hypothesized that: (1) Vegetation carbon density would decrease with increasing degradation, and (2) Soil carbon density would decrease with increasing degradation. Our study reveals changes in vegetation and soil carbon density in degraded grasslands in northwest Liaoning and provides fundamental theoretical support for the scientific management of carbon pools in these degraded areas.

## Results

### Carbon density of grassland vegetation with different levels of degradation

**Fig. 1:**
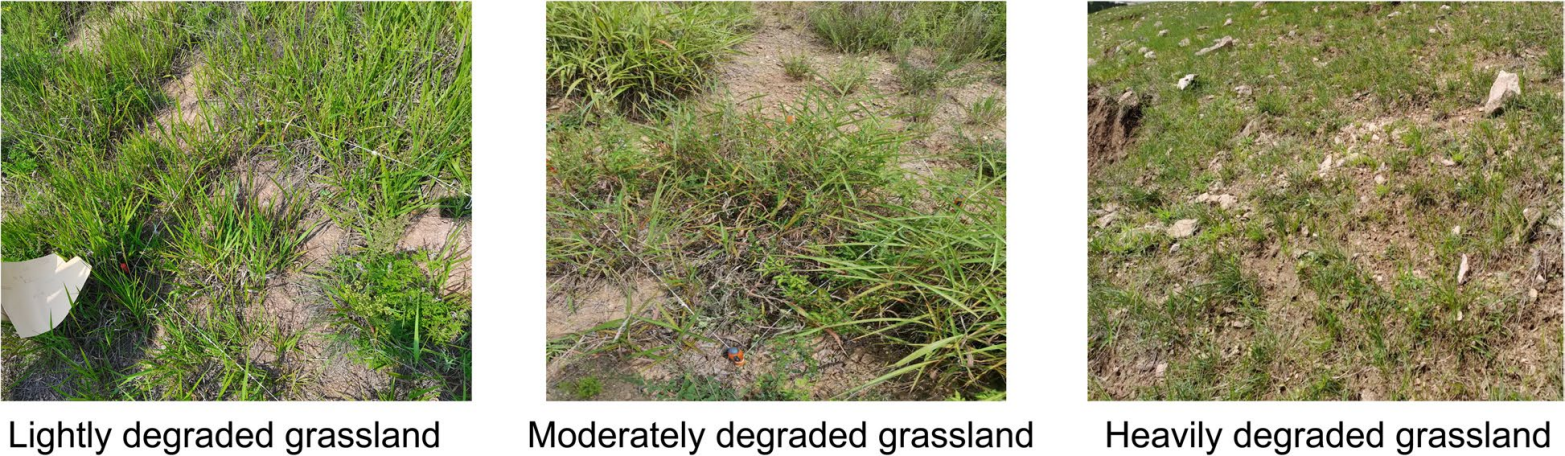
The effect of degradation on the carbon density of living plant, plant litter (**a** and **b**) and below ground vegetation (**c** and **d**). AGV: Above-ground vegetation, BGV: below-ground vegetation, LD: Light degradation, MD: Moderate degradation, SD: Severe degradation

With the intensification of degradation, both aboveground vegetation carbon density and belowground vegetation carbon density show significant differences (Table 1). Compared to mildly degraded grasslands, the above-ground living vegetation and litter carbon densities of heavily degraded grasslands decreased by 161.66 g C m^−2^and 36.87 g C m^−2^, respectively. As grassland degradation increased, the total carbon density of grassland vegetation decreased by 198.53 g C m^−2^ (Fig. 1 a, b). Below-ground vegetation carbon density, under the influence of grassland degradation, exhibited a significant decreasing trend with increasing soil depth. Moreover, for the same root depth at different degradation levels, carbon density showed a decreasing trend with the intensification of degradation, reaching its lowest value at a depth of 50–100 cm. Overall, with the intensification of degradation, below-ground vegetation carbon density significantly decreased, totaling a reduction of 952.51 g C m^−2^ (Fig. 1 c, d).^1^

**Table 1:** Effects of degradation on vegetation carbon density (*p* < 0.05)

Response variable	df	*F*	*P*
AGV	2	19.752	0.000
LP	2	17.154	0.000
PL	2	8.258	0.004
BGV	2	18.122	0.000
Depth of root system			
0-10 cm	2	9.305	0.002
10-20 cm	2	9.185	0.002
20-30 cm	2	4.654	0.027
30-50 cm	2	3.291	0.065
50-100 cm	2	8.885	0.003

**Fig. 2:**
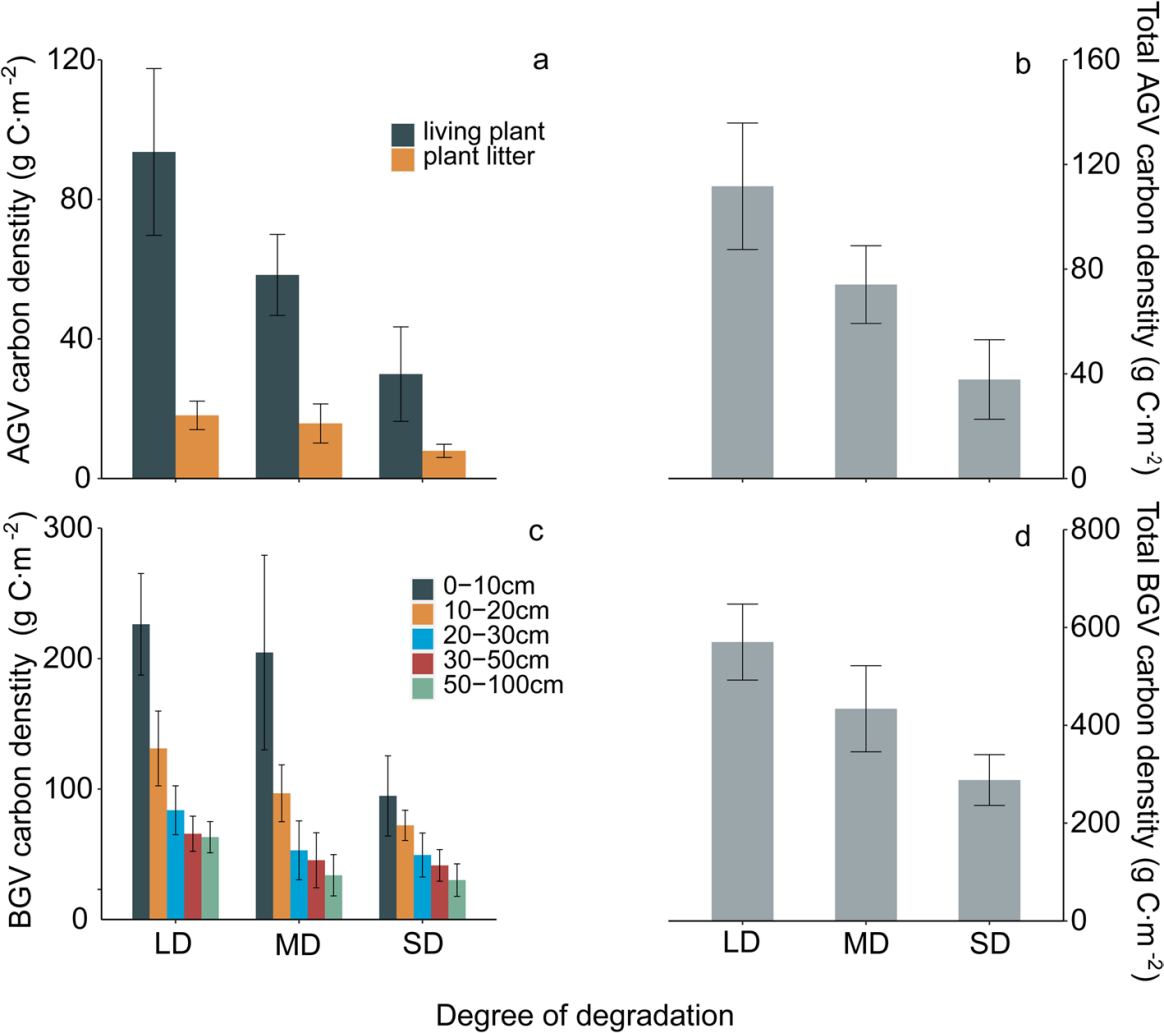
Effect of degradation on total vegetation carbon density (**a**) and the distribution of vegetation carbon (**b**). AGV: Above-ground vegetation, BGV: below-ground vegetation, LD: Light degradation, MD: Moderate degradation, SD: Severe degradation

Grassland degradation results in a significant overall decrease in vegetation carbon density. With the intensification of degradation, the total vegetation carbon density of grasslands decreased by 1151.04 g C m^−2^ (Fig. 2a). Based on the distribution of overall vegetation carbon density, underground vegetation carbon density accounts for a higher proportion than above-ground vegetation carbon density at different levels of degradation (Fig. 2b). The underground carbon density of grasslands at different levels of degradation accounts for 83%, 85%, and 88% of the total vegetation carbon density, while the above-ground carbon density accounts for 17%, 15%, and 12% of the total vegetation carbon density (Fig. 2b), indicating that underground vegetation carbon density is the main contributor to total vegetation carbon density.

### Carbon density of grassland soil with different levels of degradation

**Fig. 3:**
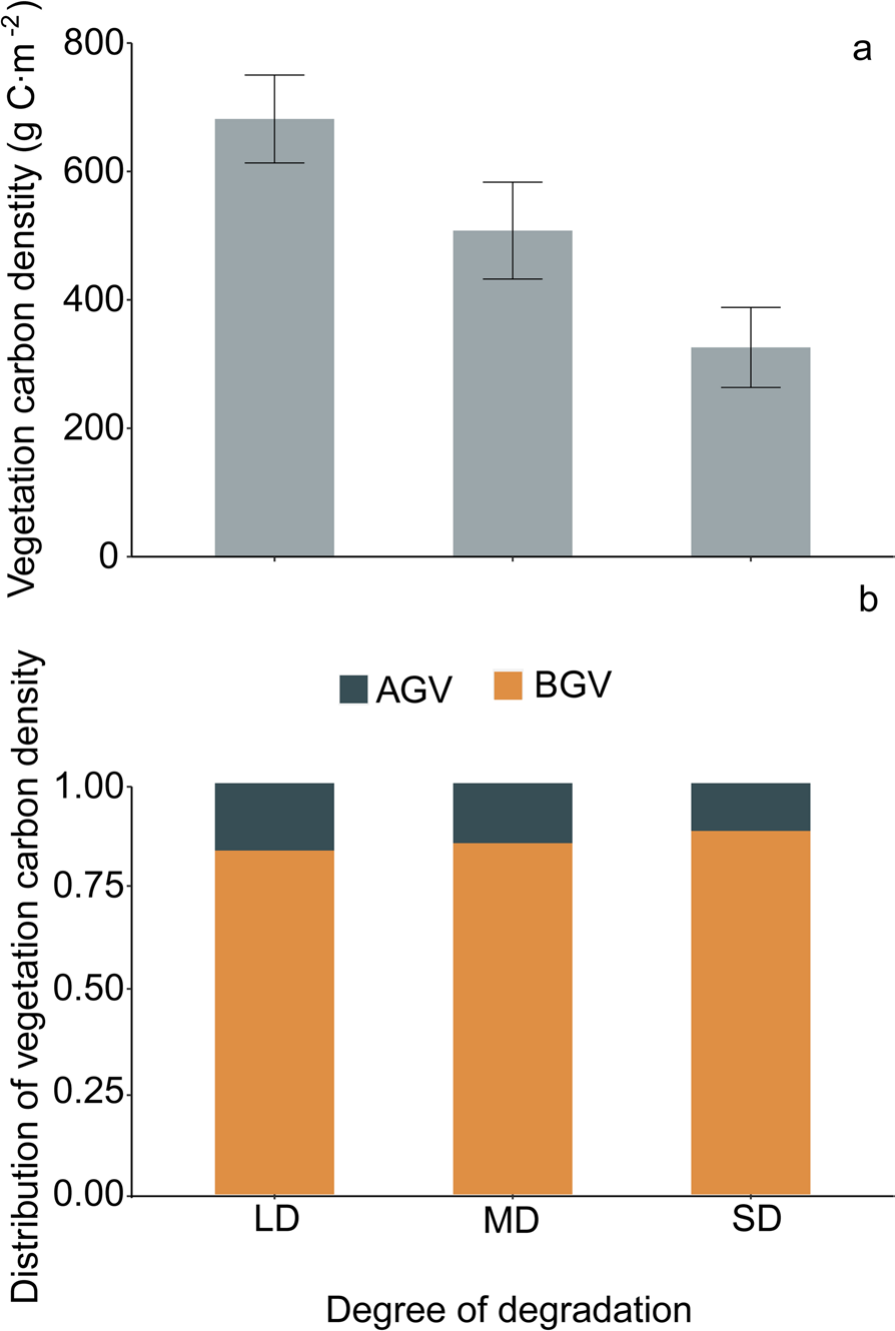
The effect of degradation on the carbon density of soil layered (**a**) and distribution of soil carbon density (**b**). LD: Light degradation, MD: Moderate degradation, SD: Severe degradation

The soil layer carbon density profile reveals that with an increasing degree of degradation, there is no significant difference in soil carbon density across various soil layers (Table 2). In terms of soil carbon density, moderate degradation exhibits a different trend compared to mild and severe degradation in grasslands. Under moderate degradation, the soil carbon density in the 0–10 cm layer is higher than that in mild and severe degradation. However, the soil carbon density in the 10–20 cm layer under moderate degradation is lower than the other two degraded grasslands (Fig. 3a). Overall, the soil carbon density distribution shows that the three levels of degraded grasslands have the highest accumulation of soil carbon density in the surface soil layer (0–10 cm, 10–20 cm), accounting for 50.36%, 45.02%, and 54.73% of the soil carbon density in different degrees of degradation, respectively. Among them, the distribution of soil carbon density in heavily degraded grasslands is the highest in the shallow soil layer (Fig. 3b).^2^

**Table 2:** Effects of degradation on soil carbon density (*p* < 0.05)

Response variable	df	*F*	*P*
ST	2	0.217	0.808
Depth of soil			
0–10 cm	2	0.538	0.595
10–20 cm	2	12.109	0.001
20–30 cm	2	0.059	0.943
30–50 cm	2	0.22	0.805
50–100 cm	2	0.045	0.956
GE	2	4.544	0.029

**Fig. 4:**
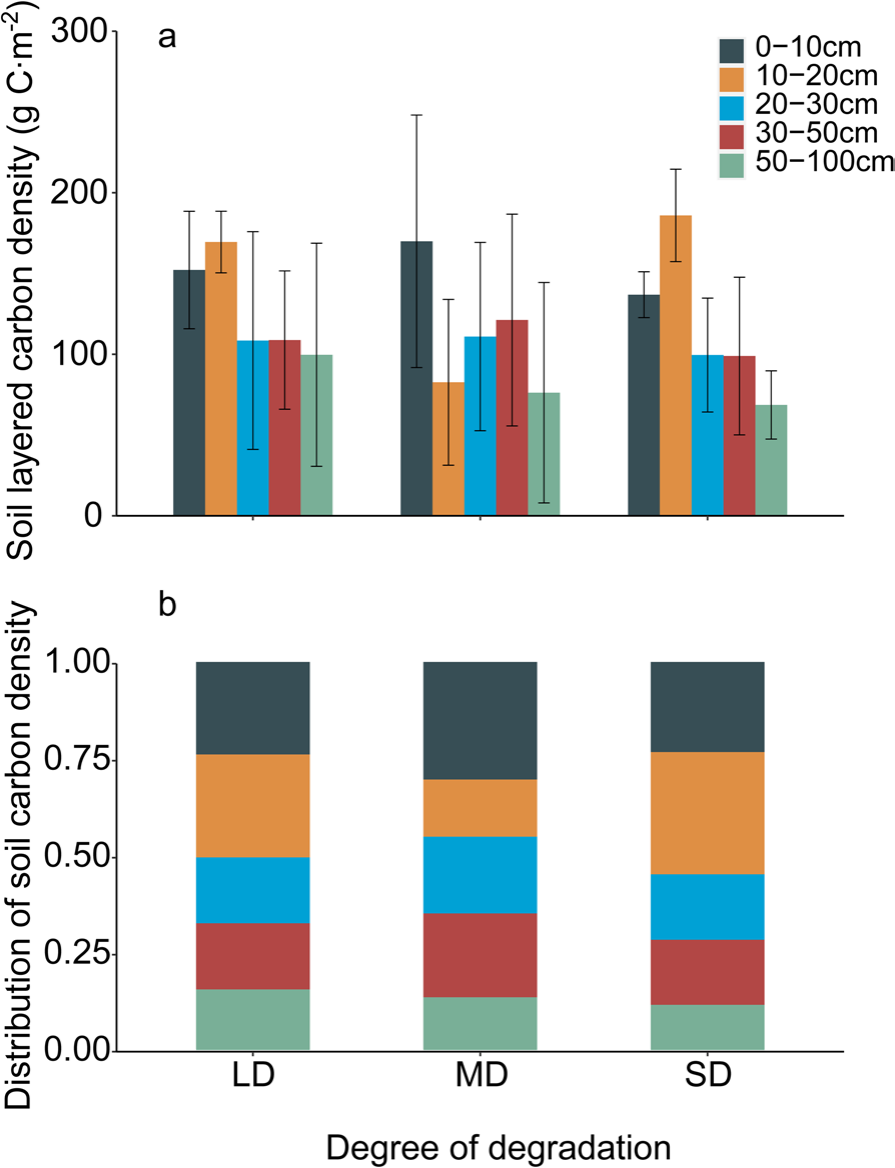
Effect of degradation on total soil carbon density. LD: Light degradation, MD: Moderate degradation, SD: Severe degradation

In summary, the values of total soil carbon density are 638.66 g C m^−2^, 560.95 g C m^−2^, and 589.89 g C m^−2^, respectively. Compared to mildly degraded grasslands, the soil carbon density of moderately and heavily degraded grasslands decreased by 77.70 g C m^−2^ and 48.78 g C m^−2^, respectively (Fig. 4). The soil carbon density of moderately degraded grasslands is slightly lower, but there is no significant difference in soil carbon density overall (Table 2).

### Carbon density of grassland with different degrees of degradation

**Fig. 5:**
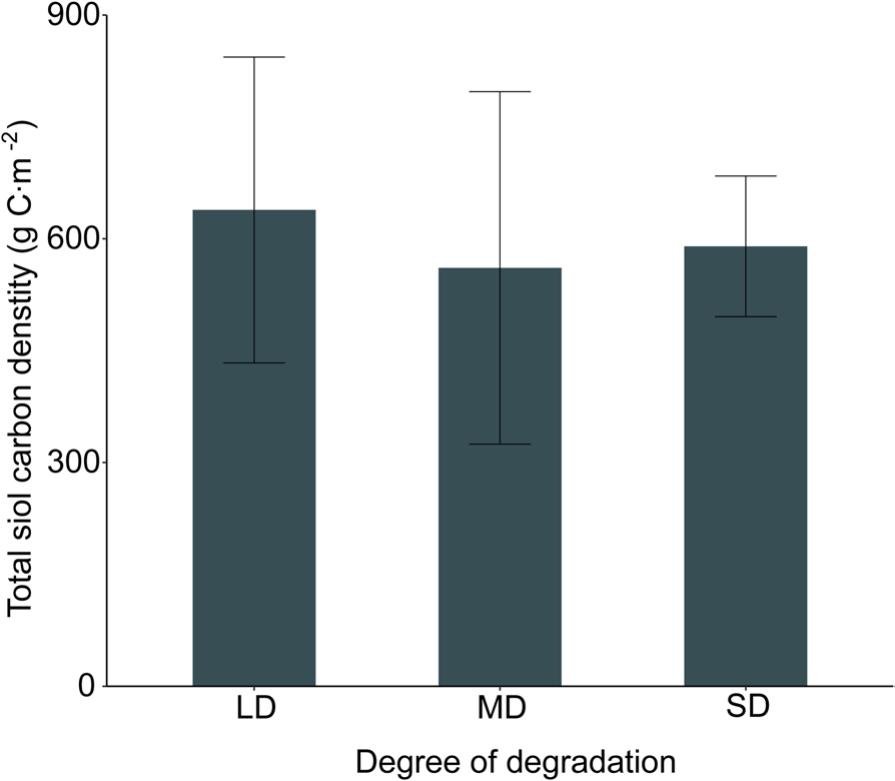
Effects of degradation on carbon density in grassland. LD: Light degradation, MD: Moderate degradation, SD: Severe degradation

The Total carbon density of grassland ecosystems exhibits significant differences across various degradation levels (Table 2). Results regarding the total carbon density in grassland ecosystems reaffirm that lightly degraded grasslands have the highest carbon density. Additionally, with increasing degradation levels, there is a declining trend in the total carbon density of the ecosystem. In heavily degraded grasslands, a substantial decrease in the total carbon density is evident, with a decrease of 416.96 g C m-2 compared to lightly degraded grasslands (Fig. 5).

## Discussion

### Vegetation carbon density

With the increasing degree of degradation, both the vegetation biomass and carbon storage in the degraded grasslands in the northwestern part of Liaoning Province are exhibiting a significant decline. This finding is consistent with the findings of many studies on degraded grasslands, which suggest that degradation negatively affects above-ground carbon stocks and reduces the carbon sequestration capacity of grassland vegetation [15, 46]. The predominant reason for this trend is the ecological vulnerability of the grasslands in northwestern Liaoning. These grasslands are adversely impacted by the erosive forces of sand and dust from Inner Mongolia, coupled with recurrent droughts and pronounced soil erosion. The pre-existing biodiversity has suffered varying degrees of disruption. The drastic reduction in vegetation biomass serves as a primary driver for the significant decrease in aboveground carbon storage [38]. Secondly, with human activities and climate change, soil moisture and nutrient effectiveness have decreased, causing further degradation and ultimately a significant decrease in vegetation biomass and carbon stocks [47]. At the same time, the increased degradation also changes the quality and quantity of litter material returned to the soil from above-ground vegetation, resulting in a decrease in the decomposition rate of above-ground vegetation litter material, and ultimately a decrease in the carbon pool of litter material [3].

As the degree of degradation intensifies, there is a continuous decline in both root biomass and its carbon storage. Moreover, a significant positive correlation is observed between the degree of degradation and the reduction in root biomass, along with a decrease in root carbon density [48, 49]. The root system is an important structure connecting plants to the soil and is a channel for nutrient uptake and transport [50]. As degradation intensifies, the main reason for the decrease in carbon density in the belowground root system is the loss of soil water and nutrients due to trampling by herbivores on degraded grasslands and sanding of grasslands, which affects root growth and decomposition of dead roots, leading to a decrease in root carbon accumulation [15]. Furthermore, as the degradation intensifies and the above-ground vegetation decreases, the area available for photosynthesis is greatly reduced and the root system has to provide nutrients to the vegetation for further growth, which affects the normal growth of the root system and its carbon accumulation [51], ultimately leading to a decrease in root carbon density as the degradation intensifies.

The study findings demonstrated that subsurface root biomass was more abundant in shallow soils and exhibited a significant reduction as soil depth increased, indicating a 'T'-shaped spatial distribution that aligns with previous research. Furthermore, the total root biomass decreased as degradation intensified [28, 51, 52]. The primary factor affecting the carbon density of below-ground vegetation is root biomass. Due to the unique distribution characteristics, the primary reason for the decrease in root carbon density with increasing soil depth is the concomitant decrease in root biomass with increasing soil depth.

### Soil carbon density

Unlike vegetation carbon density, soil carbon density does not exhibit a significant difference with increasing degradation. Soil carbon pools store a greater amount of carbon and thus dominate the carbon pool of grassland ecosystems, as compared to vegetation carbon density [23]. In our experimental site, which is primarily dominated by *Poaceae* grass *Leymus chinensis* (*L. chinensis*), the ability of *L. chinensis* to establish individuals through rhizomes makes it more adaptable than other plants. Consequently, when degradation occurs, *L. chinensis* relies on rhizome reproduction to exhibit greater resilience against above-ground disturbances than sexual propagation [18]. However, when *L. chinensis* meadows undergo degradation, there is an increase in soil permeability. Consequently, the soil carbon density in *L. chinensis* meadows does not exhibit a significant decline with the intensification of degradation. Additionally, a larger proportion of perennial grasses and sub-shrubs are present in more severely degraded grasslands [53]. These plants have a greater capacity to sequester carbon than annuals, thereby making the difference in soil carbon density in severely degraded grasslands compared to lightly and moderately degraded grasslands insignificant [54]. Moreover, due to the relatively large soil carbon stocks, their response to environmental changes in grasslands and the intensity of degradation is insignificant over a short period of time, leading to no significant differences in soil carbon density [29].However, shallow soil carbon density is sensitive to environmental disturbances and varies with the degree of degradation [52]. Generally, shallow soils are more influenced by roots and litters, which jointly contribute to carbon accumulation in shallow soils. Consequently, shallow soils have a higher carbon density compared to deep soils, as reflected in the varying degrees of degraded grassland [55]. Our study on soil carbon density in stratified layers of degraded grasslands revealed that soil carbon density in the shallow layers of heavily degraded soils (0–10 cm) was greater than in lightly and moderately degraded grasslands. This was mainly due to the fact that, under the influence of grassland degradation, plants reintroduce nutrients into the shallow soil and store them as a resource for their next revival [56]. Moreover, shallow soils become more sensitive to anthropogenic and natural disturbances as the degree of degradation increases, leading to higher carbon densities in shallow soils of moderately degraded grasslands than in mildly and highly degraded grasslands.

From the current perspective, moderate and severe degraded grasslands exhibit a decreasing trend in soil carbon density compared to lightly degraded grasslands, although the change is not statistically significant. With the further exacerbation of grassland degradation, the nutrient and water conditions in the soil environment deteriorate progressively. Ultimately, this may lead to significant losses in soil carbon density, posing a substantial threat to the carbon cycling and carbon storage in grassland soils.

### Total carbon density of grassland

In this study we found that total ecosystem carbon density showed a decreasing trend with increasing degradation, and showed significant differences. This suggests that grassland degradation has had a significant negative impact on carbon storage in grassland ecosystems in northwestern Liaoning Province.

The main reason for this is that northwest Liaoning is located at the southern edge of the Horqin sands and in a semi-arid climate zone where droughts are frequent and precipitation is generally less than evapotranspiration, leaving the grassland soils in a state of moisture deficit and leading to degradation of the grassland to varying degrees. The degradation of grasslands in northwest Liaoning has become a serious ecological problem, which eventually leads to a certain degree of damage to the carbon density of the ecosystem and poses a threat to the carbon stocks in northwest Liaoning [57, 58].

Grassland degradation is often accompanied by changes in vegetation and soils, further causing changes in ecosystem carbon stocks [59]. The impact of grassland degradation on ecosystem carbon density is mainly on vegetation carbon density, which is mainly due to the direct effect of degradation on above and below ground vegetation biomass, resulting in a decrease in litter and root biomass [60], resulting in a decrease in carbon density of grassland ecosystems in northwest Liaoning as degradation intensifies. However, the effect of degradation on soil carbon density is not obvious, the soil carbon cycle is relatively complex and many studies have shown that soil carbon fluxes in grassland ecosystems are greater than those of living vegetation [61, 62]. However, in comparison to lightly degraded grasslands, both moderately and severely degraded grasslands are already experiencing a decline in soil carbon density, emphasizing the significant issue of soil carbon storage resulting from degradation.

Overall, grassland degradation has led to a decrease in carbon density in the grassland ecosystem of northern Liaoning, causing a serious negative impact on the overall carbon storage of the ecosystem in this region [63]. The findings of this research are also reflected in previous studies on carbon stock changes in degraded grasslands across different regions. The degradation of grassland not only diminishes the carbon sequestration capacity of vegetation and alters the physicochemical properties of grassland soil but also leads to a declining trend in carbon density within the grassland ecosystem [25, 64, 65]. This indicates that grassland degradation results in a reduction of carbon cycling functionality within grassland ecosystems, posing a significant threat to the carbon sink function of grasslands. Due to the regional and climatic variations in grassland degradation, there remains a lack of quantitative information regarding the impact of grassland degradation on carbon storage consumption [66]. Therefore, research on carbon stock in degraded grasslands remains crucial.

## Conclusions

This study aims to investigate the effect of degradation intensity on vegetation and soil carbon density in northwest Liaoning grassland, which is located in an ecologically vulnerable area with frequent droughts and severe soil erosion as the main causes of grassland degradation. The results show that the degradation of grassland has led to a significant reduction in carbon density in the grassland ecosystem of northwest Liaoning. The reduction in carbon density is mainly in vegetation carbon density as the degradation is most directly applied to vegetation biomass, while there is no significant decrease in soil carbon density in a short period of time as the degradation increases. These results provide a scientific reference and basic data support for the scientific management of degraded grasslands in northwest Liaoning, and also provide a basis for scientific evaluation of the impact of grassland degradation on carbon stocks in northwest Liaoning.

## Materials and methods

### Study area

The study area is situated in Jianping County, which is located in western Liaoning Province at coordinates 119°1'-120°2'E, 40°17'-42°21'N. The topography of the region can be characterized by low mountainous areas in the south, low hilly areas in the north, and riverine areas in the west. These features fall under the category of low mountainous hilly areas of western Liaoning. The longitudinal and latitudinal factors place Jianping County within the middle temperate zone, which experiences a semi-humid, semi-arid monsoonal continental climate. The mean annual temperature is approximately 7.6 °C, with a maximum temperature of around 37 °C and a minimum temperature of -36.9 °C. The region receives an average of 615.0 mm of precipitation in a year, with the majority falling in June and August. The area experiences an annual average of 2900 h of sunshine, and is prone to wind and drought in spring and autumn, with a typical wind force of 2–3 [67]. Winter brings strong north-westerly winds. The primary soil types in the area are brown, sandy, and meadow soils. The dominant vegetation comprises the perennial rhizomatous *Poaceae* grass *Leymus chinensis* (*L. chinensis*), with other prevalent species including the *Poaceae* grass *Stipa grandis* and *Themeda triandra*, *Lamiaceae* shrub *Vitex negundo* and *Rosaceae* shrub *Armeniaca sibirica*.

### Sample site selection

**Fig. 6:**
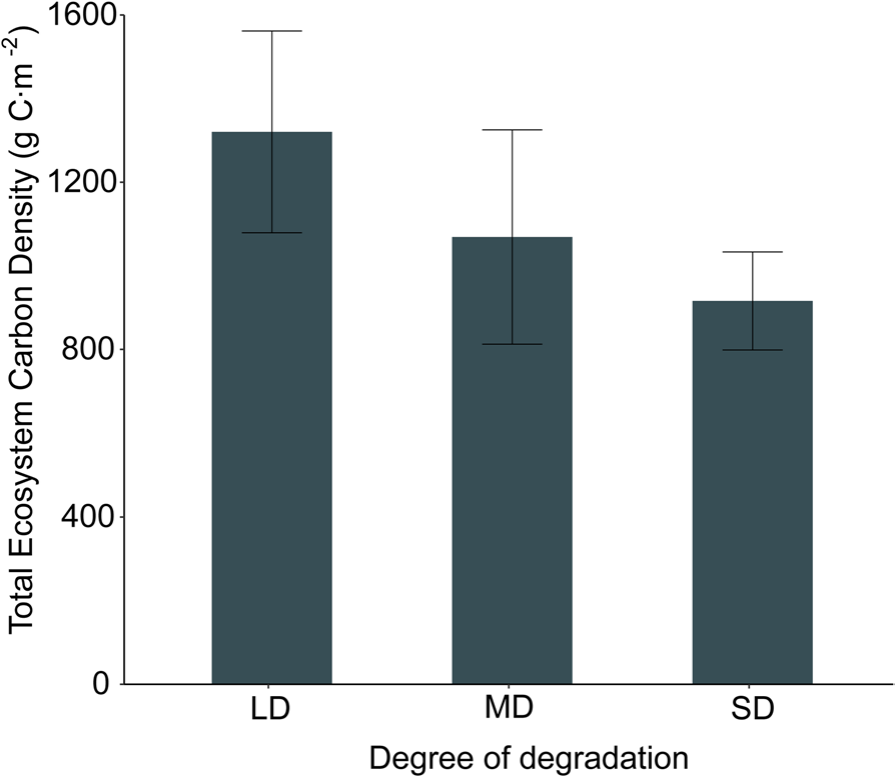
The ground surface in grasslands at different degradation intensities

The vegetation type found at the study site is *L. chinensis*. However, due to grazing and drought, the vegetation has been degraded to varying degrees. To investigate this further, three sample plots were chosen based on their degree of grazing and vegetation cover. The chosen plots have the same climatic conditions and original plant cover. The degree of degradation was categorized as lightly, moderately, and heavily degraded based on the percentage of vegetation cover. Areas with more than 70% vegetation cover were classified as lightly degraded grassland, those with 40%-60% vegetation cover were deemed as moderately degraded, and those with less than 40% vegetation cover were regarded as heavily degraded. Figure 6 illustrates images of the ground surface in grasslands at different degradation intensities (Fig. 6). Table 3 presents the biomass characteristics of both above-ground and below-ground vegetation in the sampled areas (Table 3).

**Table 3:** Vegetation characteristics under different degradation levels in northwest Liaoning Province

Degree of grassland degradation	Aboveground living biomass (g m^−2^)	Litter biomass (g m^−2^)	Belowground biomass (g m^−2^)
Light degradation	230.40 ± 40.61	63.09 ± 5.00	2058.25 ± 255.35
Moderate degradation	146.15 ± 23.37	40.56 ± 12.10	1645.90 ± 166.90
Severe degradation	68.37 ± 26.23	26.22 ± 2.58	1105.74 ± 153.49

### Experimental design and sample collection

The experiment was set up in two 100 × 100 m areas in different degrees of degraded grassland. Each area was separated by more than 1 km and three replicate plots were selected. Six replicates of each degraded grassland were set up in small plots of 1 × 1 m. The plots were used for the collection of above and below ground biomass and soil samples.

Sampling was conducted in August 2021 when plant biomass was at its peak. Aboveground biomass was measured by using scissors to cut down all living plants within the sample plot, which were then weighed and placed in envelopes. Plant litter was removed and fine sand was placed in the envelopes. The samples were then taken to the laboratory, dried at 65 °C for 48 h and reweighed. To measure below-ground biomass, soil samples were collected at five different depths (0–10 cm, 10–20 cm, 20–30 cm, 30–50 cm, and 50–100 cm) using a root auger within the same sample plot where the above-ground biomass was collected. The samples were separated into layers, placed in envelopes, taken to the laboratory, dried at 65 °C for 48 h, and reweighed. Soil samples were collected using a soil auger at the same depths as the belowground biomass sampling, with three samples taken and mixed thoroughly. The samples were taken back to the laboratory, dried naturally in the shade, and then passed through a 2 mm sieve to remove root residue. The remaining soil was ground and used for C measurement. For each soil layer, three replicates were taken using a 100 cm^3^ ring cutter and dried in a 105 °C oven to a constant weight. The samples were weighed in their dry state and used to determine the soil bulk density and gravel volume for each layer.

### Sample determination and carbon density calculation

The plant and soil samples, which had been dried to a constant weight, were crushed and sieved using an Elemental Analyser (Elemental Vario Micro, Germany) to determine their carbon and nitrogen content. Subsequently, the carbon density was calculated to obtain the vegetation and soil carbon densities.

Vegetation carbon density:

Carbon density of above-ground vegetation.

- ①* LP carbon density (g ∙ C m*^*−2*^*)* = *LP* × *VCS*
- ②* PL carbon density (g ∙ C m*^*−2*^*)* = *PL* × *VCS*

Carbon density of below-ground vegetation:

$$BGV\,carbon\,density\,(g\,Cm^{-2})=\;\sum\limits_{i=1}^n\;BGVi\;\times\;VCSi$$

where "LP" represents the above-ground living vegetation in grams, "PL" represents the above-ground litter in grams, "n" represents the number of root depth stratifications, "BGVi" represents the "i" layer of below-ground roots in grams, and "VCSi" represents the corresponding carbon content of vegetation in percentage.

Soil carbon density:

$$Soil\,carbon\,density\,(g\,Cm^{-2})=\;\sum\limits_{i=1}^n\;Di\,\times\,Bi\,\times\,(1\,-Gi)\,\times\,SCSi\,\times\,10^{4}$$

where "n" represents the number of soil layers, "Di" represents the depth of the soil layer in centimeters, "Bi" represents the soil bulk density in grams per cubic centimeter, "Gi" represents the percentage of volume in percent of gravels greater than 2 mm in diameter in layer "i", and "SCSi" represents the soil carbon content in percentage at depth "i".

### Statistical analysis

This study employed one-way analysis of variance (ANOVA) to individually assess the significance between different levels of degradation for various indicators: aboveground live vegetation carbon density, belowground vegetation carbon density, belowground vegetation stratified carbon density, total vegetation carbon density, soil carbon density, soil stratified carbon density (excluding the 0–10 cm soil layer), and total carbon density of grassland ecosystems. The ANOVA was conducted under the assumption of homogeneity of variances, and post hoc tests were performed using the Tukey Honestly Significant Difference (HSD) method. Non-parametric Kruskal–Wallis (K-W) tests were applied to examine the significance of litter carbon density and soil carbon density in the 0–10 cm soil layer across different levels of degradation, with post hoc tests using the Bonferroni method. The significance of each indicator is presented in the table. Data analysis for the entire dataset was conducted using the R software (R Core Team, 2022), and graphical representations were created using the same software.

## Data Availability

All data generated or analysed during this study are included in this published article.

## Footnotes

1 AGV: Carbon density of above-ground vegetation; LP: Live Plants; PL: Plant litter; BGV: Carbon density of below-ground vegetation.

2 ST: Soil total carbon density; GE: Carbon density of grassland ecosystem.
